# Integrative Brain States Facilitate the Expression of Parkinson's Tremor

**DOI:** 10.1002/mds.29506

**Published:** 2023-06-26

**Authors:** Michiel F. Dirkx, James M. Shine, Rick C. Helmich

**Affiliations:** ^1^ Centre for Cognitive Neuroimaging, Donders Institute for Brain, Cognition and Behaviour Radboud University Nijmegen The Netherlands; ^2^ Department of Neurology, Center of Expertise for Parkinson and Movement Disorders, Donders Institute for Brain, Cognition and Behaviour Radboud University Medical Centre Nijmegen The Netherlands; ^3^ Brain and Mind Center The University of Sydney Sydney New South Wales Australia

**Keywords:** Parkinson's disease, tremor, functional magnetic resonance imaging, cerebral integration

## Abstract

**Background:**

Parkinson's disease (PD) rest tremor emerges from pathological activity in the basal ganglia and cerebello‐thalamo‐cortical circuits. A well‐known clinical feature is the waxing and waning of PD tremor amplitude, but the mechanisms that drive this variability are unclear. Previous work has shown that arousal amplifies PD tremor by increasing between‐network connectivity. Furthermore, brain states in PD are biased toward integration rather than segregation, a pattern that is also associated with increased arousal.

**Objective:**

The aim was to test the hypothesis that fluctuations in integrative brain states and/or arousal drive spontaneous fluctuations in PD rest tremor.

**Methods:**

We compared the temporal relationship between cerebral integration, the ascending arousal system, and tremor, both during cognitive load and in the resting state. In 40 tremor‐dominant PD patients, we performed functional magnetic resonance imaging using concurrent tremor recordings and proxy measures of the ascending arousal system (pupil diameter, heart rate). We calculated whole‐brain dynamic functional connectivity and used graph theory to determine a scan‐by‐scan measure of cerebral integration, which we related to the onset of tremor episodes.

**Results:**

Fluctuations in cerebral integration were time locked to spontaneous changes in tremor amplitude: cerebral integration increased 13 seconds before tremor onset and predicted the amplitude of subsequent increases in tremor amplitude. During but not before tremor episodes, pupil diameter and heart rate increased and correlated with tremor amplitude.

**Conclusions:**

Integrative brain states are an important cerebral environment in which tremor‐related activity emerges, which is then amplified by the ascending arousal system. New treatments focused on attenuating enhanced cerebral integration in PD may reduce tremor. © 2023 The Authors. *Movement Disorders* published by Wiley Periodicals LLC on behalf of International Parkinson and Movement Disorder Society.

## 1 Introduction

Parkinson's disease (PD) is a neurodegenerative disorder characterized by bradykinesia, rigidity, and resting tremor.^1^ Whereas the presence and severity of bradykinesia and rigidity are relatively stable, this is not the case for resting tremor, which spontaneously waxes and wanes. Understanding the mechanisms that drive these spontaneous fluctuations may help develop a targeted therapy. Previous work has shown that PD tremor is produced by abnormal activity in both the basal ganglia and the cerebello‐thalamo‐cortical circuit.^2^, ^3^, ^4^, ^5^, ^6^ According to the *dimmer–switch* hypothesis, the basal ganglia act as a switch (initiating tremulous activity) and the cerebello‐thalamo‐cortical circuit acts as a dimmer (amplifying the amplitude of tremor).^4^ This hypothesis was validated using intracranial recordings.^6^ However, it remains unclear which mechanisms facilitate the enhanced coupling between basal ganglia and the cerebello‐thalamo‐cortical network. Here, we test whether dynamic changes between intrinsic brain states are linked to spontaneous fluctuations in PD tremor.

The increased coupling between distinct brain circuits in PD tremor fits with the general finding that brain states in PD are biased toward integration rather than segregation.^7^, ^8^, ^9^ For instance, PD patients have an increased dwell time in integrative brain states, and this abnormality can be (partially) restored with dopaminergic medication.^7^, ^8^, ^10^, ^11^ Furthermore, increased cerebral integration correlated with motor symptom severity^7^ and its improvement with dopaminergic medication.^10^ Recently, freezing in PD, which like tremor is a highly episodic phenomenon, has been linked to pathologically increased between‐network connectivity of the cortex, leading to impaired communication with the dopamine‐depleted basal ganglia.^12^

Both PD tremor and the expression of integrative brain states have been linked to arousal and noradrenergic mechanisms. PD tremor is amplified during cognitive load, which is accompanied by increased between‐network connectivity of the cerebello‐thalamo‐cortical circuit and a cognitive control network.^13^ Furthermore, fluctuations in pupil diameter and heart rate (ie, proxy measures of the noradrenergic ascending arousal system) were locked to fluctuations in tremor amplitude.^13^ In healthy controls, atomoxetine—a noradrenaline reuptake inhibitor—increased brain integration within the context of a cognitive task.^14^ Therefore, it has been proposed that the locus coeruleus (LC) noradrenergic system regulates the balance between cerebral segregation and integration.^15^

These findings suggest that spontaneous fluctuations in PD rest tremor may be driven by integrative brain states, increased levels of arousal, or a combination of both. We tested this hypothesis by performing resting‐state functional magnetic resonance imaging (fMRI) on 40 tremor‐dominant PD patients with concurrent recordings of tremor (using electromyography [EMG] and accelerometry) and proxy measures of the ascending arousal system (pupil diameter and heart rate). To distinguish between mechanisms that generate versus amplify tremor, we compared baseline episodes, episodes preceding tremor, and tremor episodes. Next, we investigated the temporal relationship between the measures of cerebral integration, the ascending arousal system, and the expression of tremor.

## 2 Patients and Methods

### 2.1 Study Population

We included 40 patients diagnosed with PD and a history of resting tremor. Exclusion criteria were (1) neurological comorbidity, (2) signs of psychogenic tremor, (3) allergy against levodopa‐benserazide/domperidone, and (4) cognitive impairment (Mini‐Mental State Examination <24 or Frontal Assessment Battery <18).^16^, ^17^ The study was approved by the local ethics committee; written informed consent was obtained. We included only patients who showed a clear tremor during scanning (ie, 4–6 Hz peak in the EMG power spectrum). This resulted in 33 tremor‐dominant PD patients during “cognitive load”^18^ and 34 patients during “resting state” (overlap of 30 patients), see Table 1 Note that the first cohort was also used in a previous paper.^13^ There, we reported that cognitive load amplifies PD tremor via increased cerebral connectivity between two predefined cerebral networks (cognitive control network and cerebello‐thalamo‐cortical network), using dynamic causal modelling (DCM).^13^ Here, we used a different method that focuses on whole‐brain connectivity in terms of cerebral integration versus segregation (see later). To validate this new method, we first tested whether whole‐brain integration is indeed sensitive to changes in cognitive load in PD (which amplifies tremor).^13^ Next, we tested our main research question in a different (resting‐state) data set: whether there is a systematic relationship between changes in cerebral integration and *spontaneous* fluctuations in tremor. Patients were tested in a practically defined *off* state (ie, >12 hours after levodopa, >30 hours after dopaminergic agonists, and >24 hours after β‐blockers)^19^, ^20^ and after abstention from caffeine (tea and coffee) >12 hours.

**TABLE 1:** Clinical characteristics of subjects

Characteristics	Mean (±SD)
Age	63.3 ± 3.2
Male/female	17/10
Disease duration (y)	2.9 ± 2.19
H&Y	2 (range: 1–3)
FAB	17.2 ± 0.9
MMSE	29.1 ± 1.4
MDS‐UPDRS	
Total	44.7 ± 17.4
Nontremor (B + R)	
Most	12.6 ± 4.8
Least	8.7 ± 5.1
Axial	4.6 ± 0.6
Rest tremor	
Most	4.4 ± 0.2
Least	2.5 ± 0.3
Constancy	3.6 ± 0.1

*Note*: Disease characteristics of all patients included for the resting‐state cohort (who had sufficient tremor episodes for final analyses, ie, 27 of 40) are shown (Hoehn & Yahr: median, minimum and maximum scores in parentheses; other parameters: mean, standard deviation in parentheses). Disease severity of each patient was measured using the H&Y stages (maximum is 5) and the MDS‐UPDRS, Part III (maximum score is 132). Limb rigidity is calculated as the sum of MDS‐UPDRS item 3 (excluding item “neck”), limb bradykinesia as the sum of items 4 and 8, and limb resting tremor as the sum of items 17 (excluding item “lip/jaw”) and 18. Clinical characteristics of the cognitive load cohort are reported elsewhere.^13^

Abbreviations: SD, standard deviation; H&Y, Hoehn & Yahr; FAB, Frontal Assessment Battery; MMSE, Mini‐Mental State Examination; MDS‐UPDRS, Movement Disorders Society Unified Parkinson's Disease Rating Scale.

### 2.2 Image Acquisition and Preprocessing

fMRI was performed on a 3‐T MRI system (Siemens PRISMA, Erlangen, Germany). We used a multiband echo planar imaging sequence (multiband acceleration factor = 4, repetition time = 0.859 seconds, echo time = 34 ms, 44 axial slices, voxel size = 2.2 mm isotropic, field of view = 225 mm, scanning time = ~10 min, 700 images). The first five images were discarded. High‐resolution anatomical images were acquired using a magnetization‐prepared rapid gradient‐echo sequence (repetition time = 2.300 seconds, echo time = 3.03 ms, voxel size = 1.0 mm isotropic, 192 sagittal slices, field of view = 256 mm, scanning time = ~5 minutes).

fMRI was analyzed using *SPM12* (http://www.fil.ion.ucl.ac.uk) and *FEAT* (FMRI Expert Analysis Tool) 6.00, part of *FSL* (FMRIB's Software Library, www.fmrib.ox.ac.uk/fsl). First, we used ICA‐AROMA (independent component analysis‐based automatic removal of motion artifacts) to remove noise components in an automated, observer‐independent manner.^21^ ICA‐AROMA requires a number of preprocessing steps in *FSL*: image registration, motion correction, nonbrain removal, spatial smoothing (using a Gaussian kernel of 5‐mm full‐width at half‐maximum), and grand‐mean intensity normalization.^22^ All components were visually checked and if necessary corrected. Next, output images from ICA‐AROMA were further preprocessed in *SPM12*: (1) coregistered to structural MRI, (2) normalized to Montreal Neurological Institute space using unified segmentation,^23^ and (3) spatially smoothed using a 4‐mm Gaussian kernel. We further denoised images by regressing out framewise displacement, root mean square change in bold signal from volume to volume, and white matter and CSF signal based on the CompCor strategy.^24^ Furthermore, data were filtered using a bandpass filter (0.002 Hz < f < 0.125 Hz). None of the patients showed excessive movement during scanning, which was defined as scan‐to‐scan movement that exceeded 2.2 mm (ie, the voxel size).^25^

### 2.3 Experimental Design and Behavioral Parameters

**FIG 1:**
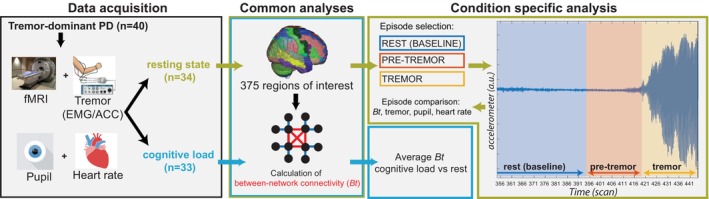
Study design showing data acquisition of fMRI (functional magnetic resonance imaging) with concurrent tremor, pupil diameter, and heart rate recordings for periods of cognitive load and resting state. Next, graph theoretical methods were used to determine between‐network connectivity (ie, cerebral integration) for both resting‐state and cognitive load trials. Finally, separate analyses were conducted for cognitive load and resting state. Notably, in the resting state a selection of rest (baseline), pre‐tremor, and tremor episodes was made. [Color figure can be viewed at wileyonlinelibrary.com]

We focused on cerebral between‐network connectivity in two separate contexts: (1) periods of cognitive load interchanged with rest and (2) resting state. First, we focused on cerebral connectivity during periods of cognitive load for which we used a block design (5 × 1 minutes rest interchanged with 5 × 1 minutes performing mental arithmetic). The details are published elsewhere.^13^ Next, we proceeded to our main research question, that is, testing the hypothesis that *spontaneous* fluctuations of resting tremor are related to cerebral integration (Fig. 1). For this, we used data where patients were instructed to rest for 10 minutes during scanning with their eyes open.

We simultaneously recorded three behavioral parameters during scanning—tremor amplitude and frequency (using EMG and accelerometry)—and two proxy measures of the ascending arousal system^26^: pupil diameter (using continuous eye tracker recordings of the left eye) and heart rate (using a pulse oximeter on the left index finger). Details of the acquisition and analyses of these parameters were similar to previous studies^13^, ^27^ and are provided in Appendix S1. We calculated the mean time course across subjects of each parameter. We also investigated the relationship between tremor and pupil diameter/heart rate to detect a possible link between tremor and the ascending arousal system. The results of this for periods of cognitive load are reported previously,^13^ where we found that there is a significant correlation between fluctuations in tremor amplitude and fluctuations in pupil diameter/heart. Here, we tested whether this relationship also exists for spontaneous fluctuations in tremor amplitude, by calculating the correlation coefficient (Pearson's *R*) between the time courses of tremor and pupil diameter/heart rate for each subject in the resting state. Subsequently we tested for significant group effects using one‐sample *t* test (2‐tailed).

### 2.4 Cerebral Between‐Network Integration

First, we extracted regional time series by calculating the first eigenvariate from 375 parcels to ensure whole‐brain coverage (333 cortical parcels using the Gordon atlas,^28^ 14 subcortical regions from Harvard–Oxford subcortical atlas, and 28 cerebellar parcels from the SUIT atlas^29^; see Appendix S1). Second, we calculated the time‐resolved functional connectivity between the 375 parcels by computing the multiplication of temporal derivative metric (MTD).^30^ The MTD is defined as the point‐wise product of temporal derivatives of pairwise time series and averaged by calculating a mean value over a temporal window. We calculated the time‐resolved functional connectivity between all 375 brain regions using the MTD within a sliding window of 25 time points (~21 seconds).^8^, ^30^ Third, the Louvain modularity algorithm was applied to the functional connectivity time series using the Brain Connectivity Toolbox.^31^ This algorithm quantifies (for each temporal window) to which extent the network may be subdivided into communities with stronger within‐community than between‐community connections. Finally, the between‐module connectivity (*B*_*T*_), which quantifies the extent to which a region connects across all modules (ie, between‐network connectivity), was calculated. To obtain a measure of whole‐brain integration, we calculated the average *B*_*T*_ across all regions.

### 2.5 Relationship between Cognitive Load and Cerebral Integration

**FIG 2:**
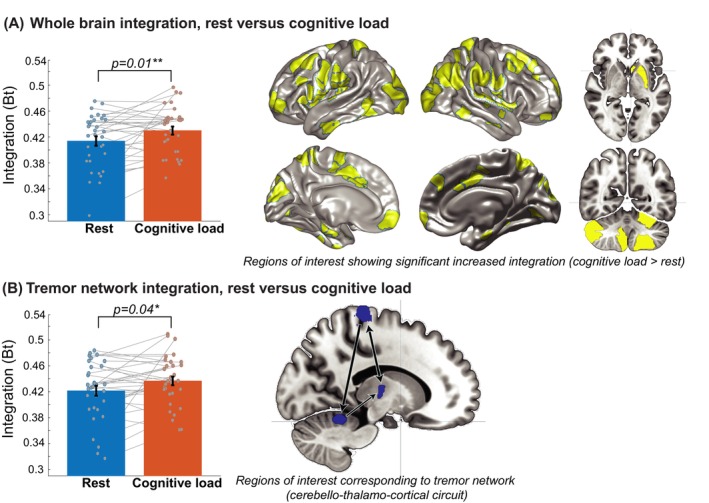
Comparison of cerebral integration between periods of rest and cognitive load from (**A**) a whole‐brain perspective and (**B**) separately for the cerebello‐thalamo‐cortical tremor circuitry, validating results from a previous study.^13^ [Color figure can be viewed at wileyonlinelibrary.com]

In the cognitive load cohort, we compared cerebral integration (*B*_*T*_) in periods of rest versus cognitive load, thereby excluding the first and last 3 seconds of each trial to remove transition effects. First, we compared whole‐brain integration by averaging *B*_*T*_ across all region of interests (ROIs) and performing a one‐sample *t* test (2‐tailed). Second, we determined exactly which ROIs showed a significant increase in integration by randomly permuting the difference in *B*_*T*_ between rest and cognitive load (iterations = 5000)—a *P*‐value of <0.05 (2‐tailed) was used to determine significance. This revealed a cognitive control network similar to previous results.^13^ Next, we calculated cerebral integration within the tremor network (ie, cerebellum, thalamus, and motor cortex; Fig. 2B) and contrasted the rest and cognitive load conditions using a one‐sample *t* test (2‐tailed).

### 2.6 Relationship between Spontaneous Tremor Fluctuations, Cerebral Integration, Heart Rate, and Pupil Diameter

In the resting‐state cohort, we investigated the temporal relationship between tremor, cerebral integration, and proxy measures of the ascending arousal system. For this, we focused on three temporal windows: (1) periods with no tremor (rest), (2) pre‐tremor, and (3) tremor. We first manually selected tremor episodes of each subject by visually inspecting the time course of tremor amplitude based on accelerometry and EMG (see Appendix S1). We used the EMG signal convolved with the hemodynamic response function for analyses related to cerebral integration (to account for the BOLD [blood oxygen level–dependent] response); for all other parameters, the unconvolved accelerometry signal was used. We defined (pre‐)tremor episodes as a fixed window of 25 scans (~21 seconds) before and after the start of tremor. This definition was based on the sliding window used for calculation of cerebral integration (25 scans) to ensure an accurate estimation. Tremor episodes that did not meet these criteria were omitted. This resulted in 60 tremor episodes over 27 subjects, with a duration of 65 ± 66 seconds (mean ± standard deviation). We had to exclude 12 and 4 subjects for pupil diameter and heart rate analyses, respectively, due to noisy data recordings. Next, we calculated the mean values of all parameters during all three temporal windows (ie, rest, pre‐tremor, and tremor) of each subject. We *z* scored all values to optimize comparison between parameters. We then calculated group averages and tested for significant differences between the three windows using a repeated‐measures one‐way analysis of variance with post hoc comparisons (least significant differences) in *SPSS* (IBM Statistics SPSS 27). Next, we inspected the temporal evolution of all parameters during pre‐tremor and tremor episodes, by calculating the mean ± standard error of the mean signal per time point. As we were interested in relative changes during (pre‐)tremor, we corrected for baseline values by subtracting the mean value of each parameter in rest. To test significant differences from baseline, we divided this signal—which was time locked to the onset of tremor episodes—into bins of two scans (~1.7 seconds) and performed a one‐sample *t* test (2‐tailed) on each bin. Finally, after these analyses showed a significant increase in whole‐brain integration prior to tremor episodes, we determined exactly which cerebral regions drove this effect by randomly permuting the difference in *B*_*T*_ between rest and pre‐tremor episodes for all 375 ROIs (5000 iterations, *P* < 0.05, Matlab R2020b).

To determine a link between tremor amplitude and integration, we calculated the relative increase in each parameter during (pre‐)tremor episodes and subsequently performed correlation analyses using Pearson's *R*. Furthermore, we determined whether fluctuations in tremor amplitude were time locked to fluctuations in pupil diameter by calculating Pearson's *R* for each subject and subsequently performing a one‐sample *t* test (2‐tailed) across the group.

## 3 Results

### 3.1 Effects of Cognitive Load on Cerebral Integration

Cognitive load significantly increased whole‐brain cerebral integration (*B*_*T*_) (*t*(32) = 2.6, *P* = 0.01; Fig. 2A). More specifically, 117 ROIs showed a significant increase in *B*_*T*_, including bilateral cortical regions from (pre)frontal, parietal, occipital, and temporal lobes and also the subcortical regions, including the thalamus and cerebellum. The cerebello‐thalamo‐cortical tremor network^3^, ^13^, ^32^ also showed increased cerebral integration (*t*(32) = 2.2, *P* = 0.04; Fig. 2B) during cognitive load versus rest. This finding validates our previous observation (on the same data set) that cognitive load (which amplifies PD tremor) is associated with increased cerebral between‐network connectivity.^13^

### 3.2 Relationship between Spontaneous Fluctuations in Tremor Amplitude, Cerebral Integration, Pupil Diameter, and Heart Rate

**FIG 3:**
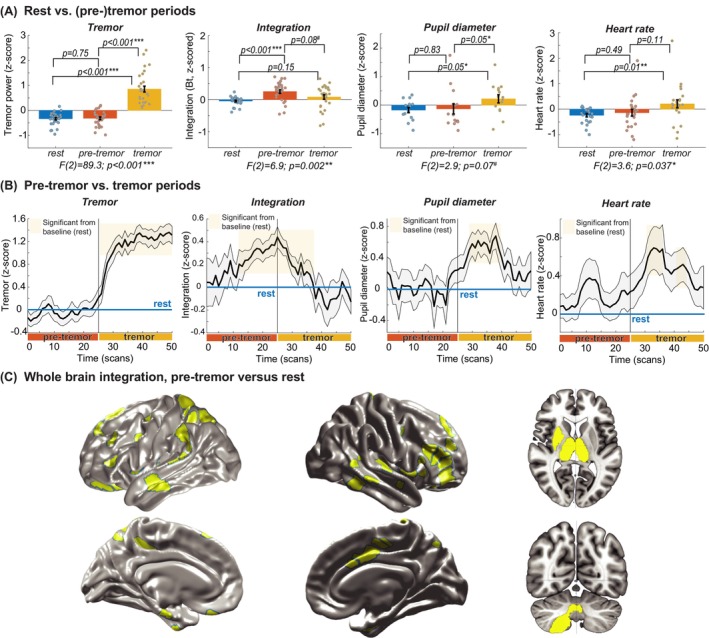
(**A**) Comparison of rest (baseline), pre‐tremor, and tremor episodes for all parameters of interest (tremor, cerebral integration, heart rate, and pupil diameter). (**B**) Temporal evolution of tremor, cerebral integration, pupil diameter, and heart rate for pre‐tremor and tremor episodes. (**C**) Regions that show significantly increased between‐network connectivity for pre‐tremor versus rest episodes. [Color figure can be viewed at wileyonlinelibrary.com]

**FIG 4:**
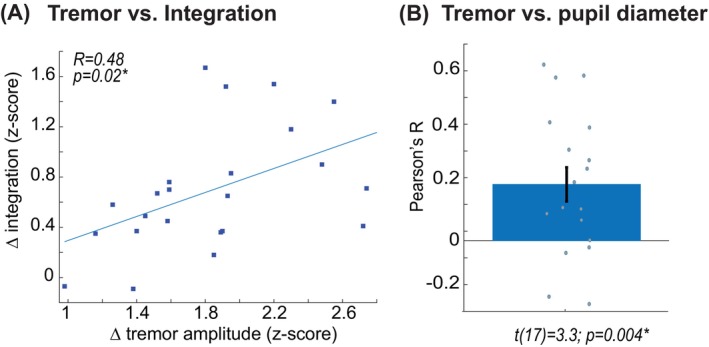
(**A**) The amount of increased integration prior to a tremor episode predicted the increase in tremor amplitude during tremor episodes. (**B**) Significant correlation of pupil diameter with fluctuations in tremor amplitude. [Color figure can be viewed at wileyonlinelibrary.com]

Cerebral integration significantly increased in the pre‐tremor episode when compared to baseline (comparison of baseline, pre‐tremor, and tremor: *F*(2, 54) = 6.9, *P* = 0.002; post hoc test on pre‐tremor versus baseline *t*(27) = 5.1, *P* < 0.001). In contrast, there was no statistical difference between baseline and pre‐tremor episodes for heart rate or pupil diameter (Fig. 3A). However, when comparing baseline to tremor episodes, there was a significant increase in heart rate (*F*(2, 44) = 3.6, *P* = 0.037; *t*(22) = 2.6, *P* = 0.01) and a trend toward significance for an increase in pupil diameter (*F*(2, 28) = 2.9, *P* = 0.07; *t*(14) = 2.1, *P* = 0.05), whereas this effect was not present for cerebral integration. This observation was confirmed when plotting the temporal evolution of parameters during (pre‐)tremor episodes (Fig. 3B), which showed that cerebral integration increases on average 13 seconds prior to tremor episodes, whereas pupil diameter and heart rate cofluctuated with tremor amplitude. These findings suggest that cerebral integration may set the stage for the occurrence of tremor episodes, whereas the arousal system subsequently modulates tremor amplitude. Therefore, the relative increase in cerebral integration during pre‐tremor episode predicted the increase in tremor amplitude during tremor episodes (Pearson's *R* = 0.48, *P* = 0.02; Fig. 4A), and spontaneous fluctuations in tremor amplitude significantly correlated with spontaneous fluctuations in pupil diameter (mean Pearson's *R* across the group: *R* = 0.2 ± 0.26; subsequent *t* test: *t*(17) = 3.3, *P* = 0.004; Fig. 4B). The relative increase in cerebral integration prior to tremor episodes was driven by 70 regions showing a significant increase in between‐network integration for pre‐tremor episodes versus baseline (Fig. 3C), which included a diffuse network consisting of frontoparietal, temporal, cerebellar, and subcortical (including bilateral thalamus) regions.

## 4 Discussion

We investigated the relationship between fluctuations in PD tremor, cerebral integration, and (proxy measures of) the ascending arousal system to test whether integrative brain states drive the expression of tremor. First, we validated previous results showing that during periods of cognitive load (when tremor is amplified) there is increased cerebral integration between brain regions related to cognitive control and tremor. Second and most important, we showed that spontaneous fluctuations in tremor amplitude are *preceded* by increased cerebral integration, which started 13 seconds before tremor onset and which predicted the amplitude of the subsequent tremor episode. In contrast, measures of arousal (pupil diameter and heart rate) were not present in the period before tremor onset, but they increased simultaneously with tremor power. These results point to two different cerebral mechanisms associated with tremor: integrative brain states create a cerebral context in which the occurrence of tremor and arousal is facilitated, whereas noradrenergic activity further amplifies tremor power.

### 4.1 Cerebral Integration and Tremor

We investigated between‐network connectivity from a whole‐brain perspective and confirmed that cognitive load (which amplifies tremor) is accompanied by increased between‐network connectivity.^13^ In particular, a diffuse number of regions from (pre)frontal, parietal, occipital, and temporal lobes, but also subcortical regions (including thalamus) and cerebellum, become more entangled during cognitive load. This fits previous studies that show that increased cerebral integration is associated with heightened information processing during cognitive performance.^33^, ^34^ In contrast, specialized programs (eg, sensorimotor learning) may benefit from functional segregation of effector‐specific regions.^35^, ^36^ This balance between functional integration and segregation is likely to be crucial for optimization of task performance,^15^ and many studies show that this balance is disturbed in PD. For example, PD patients showed a significant increase in dwell time in a state of integration, which correlated with severity of motor symptoms.^7^ Other studies confirm increased integration in PD in the hypodopaminergic state^8^ but suggest that it is a compensation mechanism. However, not all pieces of evidence point in the same direction: functional segregation has also been described as a feature of PD, although this seems to be related to nonmotor symptoms (ie, cognitive deficits).^11^, ^37^ Here, we show that PD tremor is related to increased periods of cerebral integration during periods of cognitive load and also at rest. Interestingly, in the resting state, cerebral integration preceded tremor episodes, and the amount of integration predicted the amount of tremor. Although we cannot directly infer on causality, the temporal order of events suggests that a cerebral state of integration enables the generation of tremor.

There are several possibilities in which increased cerebral integration may facilitate tremor. One possibility aligns with a previous work in freezing of gait in PD. There, freezing of gait was associated with increased cross talk between cortical motor, limbic, and cognitive networks.^12^ It was proposed that increased functional integration of these networks may lead to competing inputs onto the basal ganglia, leading to increased inhibitory output of the internal pallidum, thereby triggering freezing. The same mechanism may apply to tremor, where increased pallidal activity has also been associated with the onset of tremor.^3^, ^32^ Another possibility is that during periods of increased cerebral integration the basal ganglia and cerebello‐thalamo‐cortical circuit become more interconnected, facilitating the transmission of abnormal signs from the basal ganglia toward the cerebello‐thalamo‐cortical circuit. Therefore, we found a nonsignificant increase in functional connectivity between pallidum and motor cortex in the pre‐tremor versus rest episodes. A final possibility comes from the observation that during periods of cognitive load, a cognitive control network amplifies tremor‐related activity within the cerebello‐thalamo‐cortical circuit via excitatory influences onto the thalamus.^13^ Similarly, integrative brain states may be associated with an excitatory mode of the thalamus, enabling amplification of subthreshold tremor oscillations. Indeed, our data showed that the bilateral thalamus was among the regions showing the greatest increase in whole‐brain integration prior to tremor onset. Interestingly, it was previously described that increased thalamocortical connectivity is associated with enhanced thalamic excitability,^38^ and thalamic inhibition (eg, through dopamine^2^ or deep brain stimulation^39^) is able to suppress tremor. Thus, increased thalamic excitation, possibly mediated via integrative brain states, may play an important role in the generation of tremor. Future neurophysiological studies with deep recordings may focus on the question how exactly increased cerebral integration may set the stage for tremor.

### 4.2 Arousal and Tremor

The data show that spontaneous fluctuations in tremor are tightly linked to fluctuations of arousal as measured by pupil diameter and heart rate. Arousal is controlled by neuromodulatory systems of the brainstem, especially the LC,^40^ which have widespread projections to several (sub)cortical regions. Previous studies show that pupil diameter and heart rate are clear markers of LC activity.^41^, ^42^, ^43^, ^44^ In particular, it was shown that pupil diameter correlates with direct LC recordings in monkeys^45^ and with LC BOLD activity in humans.^26^ Furthermore, although other neurotransmitters can influence pupil diameter, heart rate is specifically controlled by the (nor)adrenergic system.

The observation that tremor is tightly linked to levels of arousal supports and extends a growing literature showing that noradrenaline plays a crucial role in the pathophysiology of tremor.^46^ For example, intravenous injection of adrenaline increases PD tremor,^47^ and this effect can be removed using β‐blockers.^48^ Furthermore, recent tracer studies using the 11‐C‐MeNER ligand show that noradrenergic neurons in LC and thalamus are relatively preserved in tremor‐dominant PD patients,^49^, ^50^ which fits an earlier postmortem study showing less degeneration of the LC in tremor‐dominant patients.^51^ In a previous study we showed that during periods of cognitive load, the LC‐noradrenergic system may amplify tremor via direct stimulation of tremor‐related activity of the thalamus.^13^ Importantly, the LC sends noradrenergic projections to all nodes of the cerebello‐thalamo‐cortical circuit,^52^ especially the thalamus.^53^ Although the almost‐immediate temporal relationship between pupil dilation and tremor suggests a modulatory role of the LC‐noradrenergic system in amplifying tremor, it does not explain why tremor emerges in the first place. Our data suggest that an increased state of cerebral integration—which precedes tremor episodes—may play a role in the emergence of tremor.

### 4.3 Arousal and Cerebral Integration

Another outstanding question is which mechanisms underlie the fluctuations in cerebral integration. Previous work suggests that this may be facilitated by the LC‐noradrenergic system, which promotes integrative capacities such as cognition and attention.^33^, ^41^ Several studies confirm the relationship with arousal and cerebral integration: admission of atomoxetine (which inhibits tonic LC activity) leads to increased cerebral segregation,^14^ and increased neural gain (supposedly mediated by noradrenaline) supports a pro‐integration network topology.^54^ Furthermore, it was recently proposed that increased cerebral integration during anxiety‐induced freezing of gait in PD is mediated via increased levels of arousal.^12^ This begs the question whether increased cerebral integration prior to tremor episodes is also mediated by the ascending arousal system. Our data show that cerebral integration starts to increase 13 seconds (on average) before the start of tremor episodes, whereas fluctuations in pupil diameter and heart rate are tightly locked to fluctuations in tremor amplitude. This suggests that increased cerebral integration is one of the first manifestations of arousal, whereas the ascending noradrenergic system kicks in only at a later stage, and that both phenomena are crucial for the emergence and expression of PD tremor. What triggers the episodes of increased cerebral integration, and how this in turn may facilitate the emergence of tremor‐related activity, remains a topic for future research.

### 4.4 Limitations

Due to the difference in temporal resolution, it is difficult to make formal statements about whether one signal (increased cerebral integration) really causes the other signal (increased tremor power or pupil dilation). Future studies may test whether interventions focused on arousal and/or cerebral integration (eg, admission of a β‐blocker) influence tremor.

## 5 Conclusion

Tremor episodes in PD are preceded by a state of increased cerebral integration, which may facilitate the emergence of cerebral tremor‐related activity. In turn, fluctuations in tremor amplitude are tightly locked to fluctuations in arousal, suggesting that arousal has a modulatory role in the production of tremor amplitude. These findings suggest that interventions aimed at attenuating cerebral integration and/or arousal (eg, via β‐blockers or biofeedback cognitive paradigms) may be effective in treating PD resting tremor.

## Author Roles

1. M.D. collected data, analyzed data, and wrote a substantial part of the paper.
2. M.S. contributed to the study design, analysis, and writing of the paper.
3. R.H. contributed to the study design and analysis and wrote a substantial part of the paper.

## Full financial disclosures for the previous 12 months

M.D. received honoraria from the Movement Disorders Society Quebec and received a grant from ParkinsonNL (P2023‐14). R.H. has served as consultant for UCB in the past 12 months and received grants from ParkinsonNL (p2023‐14), Netherlands Organization for Scientific Research (09150172010044), EU‐JPND (10510062110006), and The Michael J. Fox Foundation (MJFF‐021001). M.S. received a Viertel/Bellberry Fellowship, NHMRC GNT1193857.

## Supporting information


**Appendix S1.** Supporting Information.

## Data Availability

Once published all (pseudononymized) data will become freely available at the Donders Repository (https://data.donders.ru.nl/?1).
