# Supplementary material for: Integrative Brain States Facilitate the Expression of Parkinson's Tremor
Source: Mov Disord. 2023 Jun 26;38(9):1615–24. doi: 10.1002/mds.29506 (PMC10947311; doi:10.1002/mds.29506)
Supplement: Supplementary file 1 — Appendix S1. Supporting Information. [file MDS-38-1615-s001.docx]

**Supplement 1 – acquisition and processing of behavioural parameters during scanning**

We simultaneously recorded three behavioural parameters during scanning: tremor and two proxy measures of the ascending arousal system, which likely includes noradrenergic afferents: pupil diameter and heart rate.^1,2^

Tremor was recorded during scanning using electromyography (EMG) of the most-affected forearm muscles (extensor digitorum communis and flexor carpi radialis muscles) and accelerometry (ACC) on the dorsum of the hand using an MRI-compatible EMG and tri-axial accelerometer (Brain Products; sampling frequency (Fs) = 5000Hz). The EMG signal was analyzed using similar procedures as before.^3,4^ Preprocessing included (1) removal of MRI-induced artifacts (using the EMG-fMRI artifact reduction for motion toolbox (FARM))^5^; (2) rectification to capture the frequency of muscle bursts (3) high-pass filtering >1Hz to remove slow-frequency drifts (using FieldTrip).^6^ We calculated the first principle component of the accelerometer signal.^7^ Next, for both the EMG and ACC signal we used FieldTrip to calculate time-frequency representations between 2-8 Hz in steps of 0.001s using a 2s Hanning taper, resulting in a 0.5 Hz spectral resolution. For each patient, we calculated the time-course of the EMG and ACC at each subject’s individual tremor frequency ±1.5 Hz (mean+standard error of mean (SEM): 4.6±0.2Hz). This resulted in patient-specific regressors describing fluctuations in tremor amplitude. To remove outliers, data was logarithmically transformed and z-normalized within subjects.

To acquire a proxy measure of arousal and locus coeruleus activity^1^ pupil size (arbitrary units) was recorded continuously from the left eye via the head coil mirror using an iView X eyetracker (SMI, Needham, USA; Fs=50Hz). To create regressors describing pupil diameter over time we used several steps based on a previous study.^8^ Specifically, we first did a quality check of the data and only selected patients who had less than 25% drop in eyetracking (i.e. pupil diameter <5 pixels; n=15). Next, we divided the duration of the measurement in epochs corresponding to each scan (0.859s). Epochs in which >40% of the samples showed either a drop in eyetracking or high variability (any sample ±3SD outside the epoch mean) were discarded (proportion discarded: 6.81±5.8%). For the remaining epochs the mean value was calculated and used to build a regressor with the same temporal resolution as the scanner repetition time. Discarded epochs were interpolated across adjacent clean epochs.

To acquire an additional proxy measure of (nor)adrenergic activity we recorded heart rate using a pulse oximeter (Fs=5000Hz) around the patients left index finger. Raw pulse data were processed offline using the in-house Heart Rate Analysis toolbox (HERA) implemented in matlab for interactive visual artifact correction, peak detection and subsequent creation of regressors describing heart rate.^9^ Four patients were excluded due to noisy recordings.

**Supplement 2 – calculation of between-network connectivity**

First, we extracted regional time series by calculating the first eigenvariate from 375 parcels to ensure whole brain coverage: 333 cortical parcels using the Gordon atlas (161 and 162 regions from the left and right hemisphere respectively^27^; 14 subcortical regions from Harvard-Oxford subcortical atlas (bilateral thalamus, caudate, putamen, ventral striatum, globus pallidus, amygdala and hippocampus; <http://fsl/fmrib.ox.ac.uk>); and 28 cerebellar parcels from the SUIT atlas^28^; for each participant in the study.

Second, we calculated the time-resolved functional connectivity between the 375 parcels by computing the multiplication of temporal derivatives metric (MTD).^29^ The MTD is defined as the point-wise product of temporal derivative of pairwise time series and averaged by calculating a mean value over a temporal window (equation 1). Here, we calculated the time-resolved functional connectivity between all 375 brain regions using the MTD within a sliding temporal window of 25 time points (~21s), which provides a good trade-off between the ability to resolve the dynamics of functional connectivity and the quality of the correlation matrix estimation.^9,29^ Importantly, changing the window within ±10 time points did not significantly alter our results. Individual functional connectivity matrices were then calculated within each temporal window, thus generating a weighted 3D adjacency matrix (region x region x time) for each participant.

$MTD_{ijt}=\frac{1}{w}\sum_{t-\frac{w}{2}}^{t+\frac{w}{2}} \frac{(dt_{it} \times dt_{jt})}{\sigma_{dti} \times\sigma_{dtj}}$ (1)

Formula to determine time-resolved connectivity between pairs of regions*,* where for each time point “t” the MTD for the pairwise interaction between region *i* and *j* is defined. *Dt* is the first temporal derivative of the i^th^ or j^th^ time series at time *t,* $\sigma$ is the standard deviation of the temporal derivative time series for region *i* or *j* and *w* is the window length of the simple moving average.

Third, the Louvain modularity algorithm was applied to the functional connectivity time series using the Brain Connectivity Toolbox.^30^ This algorithm iteratively maximizes the modularity statistic, Q, for different community assignments until the maximum possible score of Q has been obtained (Equation 2). This yields a quantification of the extent to which the network may be subdivided into communities with stronger within-module than between-module connection.

$Q_{T}=\frac{1}{v^{+}}\sum_{ij} \left( w_{ij}^{+}-e_{ij}^{+} \right)\delta_{M_{i}M_{j}}-\frac{1}{v^{+}+v^{-}}\sum_{ij} \left( w_{ij}^{-}-e_{ij}^{-} \right)\delta M_{i}M_{j}$ (2)

Where *v* is the total weight of the network (sum of all negative and positive connections), *w_ij_* is the weighted and signed connection between regions *i* and *j*, e_ij_ is the strength of a connection divided by the total weight of the network, and $\delta_{M_{i}M_{j}}$ is set to 1 when regions are in the same community and 0 otherwise. The plus and minus sign symbols denote all positive and negative connections respectively. For each temporal window, regional community assignment was assessed 500 times and a consensus partition was identified using a fine-tuning algorithm from the Brain Connectivity Toolbox. The γ-parameter was set to 1. This resulted an estimate of both the time resolved modularity (*Q_T_*) and cluster assignment (*Ci_T_*) within each temporal window for each participant in the study.

Last, the between-module connectivity (*B_T_*) was calculated which quantifies the extent to which a region connects across all modules (equation 3)

$B_{iT}=1-\sum_{s=1}^{n_{M}} \left( \frac{k_{isT}}{k_{iT}} \right)^{2}$ (3)

Where $k_{isT}$ is the strength of the positive connections of region *i* to regions in module *s* at time *T*, and $k_{iT}$ is the sum of strengths of all positive connections of region *i* at time *T*. This means that the *B_T_* ranges from 0 to 1, where 1 indicates a region is uniformly distributed among all modules (integration) and 0 if all of its links are limited to its own module (segregation). To obtain a measure of whole brain integration we calculated the average *B_T_* across all regions.

**References**

1. Murphy, P. R., Robertson, I. H., Balsters, J. H. & O’Connell R, G. Pupillometry and P3 index the locus coeruleus-noradrenergic arousal function in humans. *Psychophysiology* **48**, 1532–1543 (2011).

2. Boron, W. F. & Boulpaep, E. L. *Medical Physioloy*. (Elsevier Inc., 2012).

3. Dirkx, M. F. *et al.* The cerebral network of parkinson’s tremor: An effective connectivity fMRI study. *J. Neurosci.* **36**, (2016).

4. Dirkx, M. F. *et al.* Dopamine controls Parkinson’s tremor by inhibiting the cerebellar thalamus. *Brain* **140**, 721–734 (2017).

5. van der Meer, J. N., Tijssen, M. A., Bour, L. J., van Rootselaar, A. F. & Nederveen, A. J. Robust EMG-fMRI artifact reduction for motion (FARM). *Clin Neurophysiol* **121**, 766–776 (2010).

6. Oostenveld, R., Fries, P., Maris, E. & Schoffelen, J. M. FieldTrip: Open source software for advanced analysis of MEG, EEG, and invasive electrophysiological data. *Comput Intell Neurosci* **2011**, 156869 (2011).

7. Brittain, J. S. *et al.* Distinguishing the central drive to tremor in Parkinson’s disease and essential tremor. *J Neurosci* **35**, 795–806 (2015).

8. Murphy, P. R., O’Connell, R. G., O’Sullivan, M., Robertson, I. H. & Balsters, J. H. Pupil diameter covaries with BOLD activity in human locus coeruleus. *Hum Brain Mapp* **35**, 4140–4154 (2014).

9. van Buuren, M. *et al.* Cardiorespiratory effects on default-mode network activity as measured with fMRI. *Hum Brain Mapp* **30**, 3031–3042 (2009).
